# Healthcare Ethics During a Pandemic

**DOI:** 10.5811/westjem.2020.4.47549

**Published:** 2020-04-13

**Authors:** Kenneth V. Iserson

**Affiliations:** University of Arizona, Department of Emergency Medicine, Tucson, Arizona

## Abstract

As clinicians and support personnel struggle with their responsibilities to treat during the current COVID-19 pandemic, several ethical issues have emerged. Will healthcare workers and support staff fulfill their duty to treat in the face of high risks? Will institutional and government leaders at all levels do the right things to help alleviate healthcare workers risks and fears? Will physicians be willing to make hard, resource-allocation decisions if they cannot first husband or improvise alternatives?

With our healthcare facilities and governments unprepared for this inevitable disaster, front-line doctors, advanced providers, nurses, EMS, and support personnel struggle with acute shortages of equipment—both to treat patients and protect themselves. With their personal and possibly their family’s lives and health at risk, they must weigh the option of continuing to work or retreat to safety. This decision, made daily, is based on professional and personal values, how they perceive existing risks—including available protective measures, and their perception of the level and transparency of information they receive. Often, while clinicians get this information, support personnel do not, leading to absenteeism and deteriorating healthcare services. Leadership can use good risk communication (complete, widely transmitted, and transparent) to align healthcare workers’ risk perceptions with reality. They also can address the common problems healthcare workers must overcome to continue working (ie, risk mitigation techniques). Physicians, if they cannot sufficiently husband or improvise lifesaving resources, will have to face difficult triage decisions. Ideally, they will use a predetermined plan, probably based on the principles of Utilitarianism (maximizing the greatest good) and derived from professional and community input. Unfortunately, none of these plans is optimal.

## INTRODUCTION

Disasters recur on a regular basis. In any disaster, and especially in those caused by disease, the public expects healthcare professionals to be on the front lines. Indeed, most healthcare professionals expect that of themselves and their colleagues. In most disasters, and certainly during the current COVID-19 pandemic, frontline healthcare professionals face two key ethical issues: (1) whether to respond despite the risks involved; and (2) how to distribute scarce, lifesaving medical resources. In this paper, I discuss how healthcare professionals weigh risk factors related to their response and the actions the healthcare community can take, including proper communication and mitigating responder concerns, to maximize and maintain our caregiver workforce. I then very briefly discuss the ethics of scarce resources and suggest options, such as recalling retired clinicians to service, improvisation, and husbanding available resources to mitigate rationing.

## ETHICAL ASPECTS OF THE CURRENT PANDEMIC

As COVID-19 devastates the world, bringing another feared and inevitable highly infectious pandemic to the current generation of healthcare professionals, we face a slew of ethical dilemmas. Some of our colleagues around the globe reportedly have already had to make resource-allocation decisions about which patients to treat. Others have had to struggle to provide lesser degrees of (“degraded”) care. We have little direct control over these situations. Most of the world failed to recognize the existential threat of this new coronavirus early enough to fully prepare institutional, local, regional, national, and international mobilization and response. Political expediency, hubris, scientifically ignorant leaders, and incomplete information led to this inadequate advance planning by minimizing the threat when it appeared, further delaying vital public health action.

At this point, the most vital ethical decision in our war against an unseen enemy is the one over which each of us has direct control: Will we stay to help in the fight?

Most disaster plans depend on physicians, nurses, support staff, and prehospital personnel to maintain healthcare’s frontlines during crises. Yet planners cannot automatically assume that all healthcare workers will respond. Will our hospitals and clinics have enough physicians, advanced practitioners, nurses, technicians, maintenance, and administrative staff to keep the doors open, the computers running, the linens clean, the lights on, and the facilities safe? Will our 9-1-1 systems still be able to dispatch medics, firefighters, and police? That depends on the iterative, possibly hourly or daily, decisions that each affected individual repeatedly makes.

Such decisions are not purely ethical, but rather are complex determinations based on religious and personal values, family and community responsibilities, health and financial stability, and risk assessment. In 2001, for example, the AMA Code of Ethics was modified from “solemnly commit[ing] ourselves to apply our knowledge and skills when needed, though doing so may put us at risk”^1^ to “physicians should balance immediate benefits to individual patients with ability to care for patients in the future.”^2^ The American College of Emergency Physicians, meanwhile, stated in its 2017 Code of Ethics for Emergency Physicians: “Courage is the ability to carry out one’s obligations despite personal risk or danger. Emergency physicians exhibit courage when they assume personal risk to provide steadfast care for all emergency patients, including those who are agitated, violent, infectious, and the like.”^3^

Despite these professional ethical codes, nothing—either morally or legally—compels a response to risk-prone situations. Other than military personnel, no one is required to respond to potentially life-threatening emergencies. Professional oaths and codes may serve to guide practitioners, but they are not absolutes. The factors that guide people to respond are very personal; healthcare workers’ individual behavior and that of our organizational, professional and political leadership can modify those factors to increase the number that are willing to respond.^4^

## VALUES

The moral backbone of medical professionals—a duty to put the needs of patients first—may be tested as they determine whether to stay and carry out their professional roles or to step back and decrease their own personal risks. Whether providers will stay depends on their *own risk assessment and value system*. The “duty to treat” when one’s health, life, or personal well-being is threatened is not absolute. In a risk-prone situation, each of us will prioritize our personal and professional values, those traits in ourselves that we consider to be our fundamental driving forces. “Most clinicians first assess the risks to our own and to our family’s life, health, and safety. We may then factor in, to varying degrees, our religious beliefs and personal motivations, all colored by elements of our personality. Next, we may consider professional factors, including the precepts in our healthcare profession’s oaths and codes, as well as other ethical and religious dicta to which we subscribe. Most clinicians will focus on their concrete professional responsibilities.”^5^ These professional factors include:

- Supporting/assuming the same risk as colleagues
- Collegial pressure/consequences of not helping
- Augmenting community welfare
- Fulfilling public expectation and trust
- Using societally underwritten special training and professional status
- Fulfilling implied consent to help those in need (social contract)

Emergency physicians may also feel that in these situations they are compelled to use their special knowledge about triage, allocation of scarce resources (eg, vaccines, prophylactic or treatment medications, or intensive care unit [ICU] ventilators), public health mandates (eg, isolation or quarantine, or mandatory vaccination), and the use of altered standards of care.^4^,^6^

## RISK ASSESSMENT AND MITIGATION

### Risk Assessment

When preparing for a disaster, planners should consider not how they expect people to respond, but rather why they are likely to respond.^7^ The risks to physicians and other healthcare providers’ *will vary* by the nature of the causative agent, the provider’s activities and underlying health, and the protections offered and used. People decide which risks to accept or to avoid based on their own perceptions of the source and quality of the information they receive.^8^,^9^ Quick, emotional impressions often precede and guide “rational” risk appraisals.^10^ Provider and population *perception of their risk from COVID-19 will probably not be congruent with reality*. In part, this will be due to the discordant messages from many senior politicians and other officials, but also will be influenced by the real-time updates in scientific knowledge about the disease, its transmission, and possible protective measures.

### Risk Communication and Mitigation

In crises, individuals must balance good information from valid media, government, and other sources to help identify the actual risks to themselves and their loved ones. Providing the best current information about the risks as well as the opportunities to assist during a crisis will help healthcare professionals make defensible decisions in disaster settings.^5^ Transparent and consistent information generates the trust necessary for both caregivers and the population to develop a reasonable risk assessment during conditions of uncertainty.^11^ Issuing incomplete or conflicting information, as was done during the first months of the COVID-19 outbreak, caused many providers to make decisions to respond based on heated emotions and inaccurate risk perceptions. People have been shown to naturally exaggerate the risk of phenomena that are unknown or “dreaded,” such as those with delayed, irreversible or manmade effects; those that have new, unknown, or unobservable risks; those that are global; and those that are “hyped” by the media.^5^

Historical precedent and the nature of the medical profession demonstrate that *we will have enough physicians and, probably, nurses* to treat patients. Other professional and non-professional staff needed to keep healthcare institutions operating may not be as willing to risk themselves. Recent history suggests that we probably will not have enough support personnel because, although they may be at as much or more risk than healthcare professionals, their personal safety is often considered as an afterthought by administrators. “An important lesson from the SARS outbreak is that, whereas most clinicians will “stay and fight,” vital support personnel, including those in materials and supply, logistics, cleaning, information technology communications, maintenance, and refuse removal, may feel no commitment to assist; moreover, they may feel undervalued, unprotected from risks, and ignored when they are omitted from vital communications.”^12^

If all the staff necessary to run medical facilities fail to receive timely, relevant and believable information, they may not respond, and the quality of available healthcare will deteriorate. Widely distributing accurate risk assessments and descriptions of protective measures for staff will encourage the maximal number of clinicians and other necessary personnel to respond to the situation. Therefore, disaster planners and managers should do everything possible to communicate the risks clearly to *all* members of the healthcare system and to provide them with as much support and security as possible.

**Figure 1:**
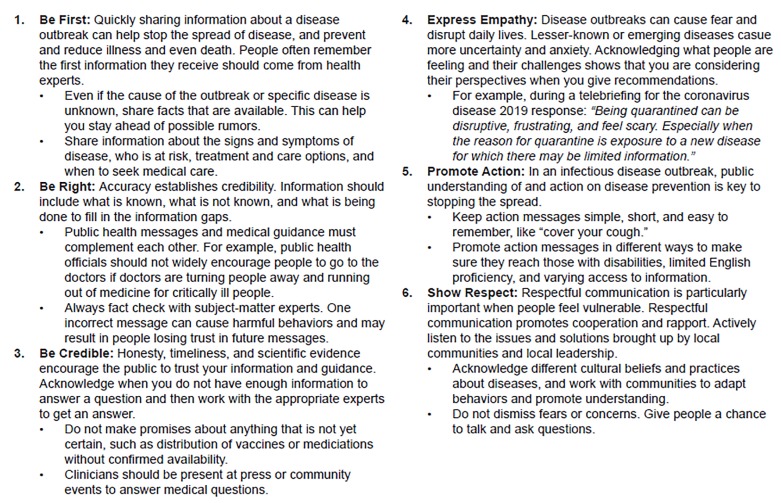
Crisis emergency risk communication in an infectious disease outbreak.^16^

Risk communication (Figure 1) is “the exchange of real-time information, advice and opinions between experts and people facing threats to their health, economic or social well-being.” 13 Its purpose “is to enable people at risk to make informed decisions to protect themselves and their loved ones.”^13^ Risk communication can help keep healthcare and other vital workers at their posts. But it must be done by appropriate people, educated in risk-communication techniques, in a trustworthy manner (honestly, frequently, open/available), and through easily accessible means, which includes role-modeling by those in charge.^14^

In addition to providing information, research shows that to attain the maximal response during risk-prone and other disasters, planners must do everything practicable to mitigate perceived risks and to address other concerns that may prevent staff from being either able or willing to work in a disaster (Table 1). To address one concern, on March 20, 2020, the American Academy of Emergency Medicine issued a position statement saying, in part, that they believe “a physician, nurse, PA, first responder or other healthcare professional has the right to be removed from the schedule of work requiring direct contact with patients potentially infected with COVID-19 for issues of personal health, such as being on immunosuppressive therapy or other similar concerns, without the risk of termination of employment.”^15^

Rarely discussed, but a key part of maintaining our workforce, is to support the psychosocial needs of the healthcare team. According to medical anthropologist Monica Schoch-Spana, “Pandemics aren’t just physical. They bring with them an almost shadow pandemic of psychological and societal injuries as well.”^17^ Psychosocial support for healthcare workers in the current war against COVID-19 will be akin to post-traumatic stress disorder treatment for soldiers manning the front lines for extended periods. People respond to the risks differently, so experienced professionals will need to intervene before tragic, adverse events occur.

## SCARCE RESOURCES AND SOME SOLUTIONS

In the current pandemic, some key resources are and will increasingly become scarce. Physicians will need to consider how to distribute available resources and obtain or improvise others. The most ethical course of action is to do everything possible to delay having to ration. Vital materials already in short supply include viral test kits and their associated equipment and reagents, personal protective equipment (PPE), ventilators, and hospital – especially ICU – beds. While China rapidly erected new, prefabricated hospitals to treat patients and many countries around the world are establishing alternative care sites, the United States has been slow to act.

Often not considered, healthcare workers, especially those with expertise treating the critically ill, will inevitably become a scarce resource. However, as the situation changes, most healthcare workers will constantly reassess their decisions about responding. As increasing numbers of personnel get sidelined due to actual or suspected disease, exhaustion, or fear for themselves or their families. Some active and retired personnel who initially stayed out of the fight or were sidelined due to illness or other circumstances may reassess their decision and join the battle. Employing senior medical students and extending advance practitioners’ scope of practice has been suggested as one way to ameliorate this problem.

In England and Wales, the National Health Service has asked about 65,000 retired doctors and nurses to return to work. In Scotland, they are recalling those who retired within the past three years. If these clinicians have been away from practice for more than a short time, they will receive brief refresher training.^26^ The Institute of Medicine, among others, have described how to best manage resource scarcity in a widespread disaster (Table 2). Many of these strategies are discussed in more detail elsewhere. 27

## ETHICS OF SCARCE RESOURCE ALLOCATION

During or after attempts at conservation, reutilization, adaption, and substitution are performed maximally, rationing will need to be implemented.^31^ The ethical principle that guides rationing is distributive justice, which requires that scarce resources be distributed fairly, providing them to those most in need. Specifically, it requires impartial and neutral decision makers to consistently apply rationing decisions across people and time (treating like cases alike).^32^ This is based on Utilitarian principles, including conservation of resources, fiduciary responsibility (stewardship), multiplier effect (does the person have a job that will save other lives?), immediate usefulness, medical success, and caretaker role.^33^,^34^ Most ethicists agree, however, that such distribution should be equitable, although in some circumstances other distribution methods, such as first come, first served; equal distribution; and even, no distribution may be more rational. Even with agreement about equitable distribution, scarcity often requires clinicians to prioritize which patients receive the resources.^33^,^34^

As the COVID-19 pandemic extends its devastation, physicians around the world are already facing the daunting task of rationing lifesaving resources. This is upending their traditional method of treating the sickest first in emergency departments or “first come first served” in the ICUs.^31^ In Italy, physicians have reported limiting ventilators to those less than 60 years old, and China and Spain have implemented medical resource rationing. The US government and many states that have developed rationing plans have yet to explicitly implement them.^35^ Many of these plans may be outdated, and none have been tested to determine whether they will save lives. In fact, a Canadian study of H1N1 patients found that 70% of patients that a rationing plan would have removed from ventilators survived with continued ventilation.^36^

Dr. Laura Evans, an intensivist at the University of Washington, is working with her state to devise a triage plan that would be doing “the most good for the most people and be fair and equitable and transparent in the process.” Yet the Washington State Health Department recently issued a statement that “triage teams under crisis conditions should consider transferring patients out of the hospital or to palliative care if the patient’s baseline functioning was marked by ‘loss of reserves in energy, physical ability, cognition and general health.’”^36^

Rationing plans must conform to general ethical principles and to existing community moral standards. Community input into these plans is vital for maintaining the public’s trust in clinicians, the institutions, and the organizations involved in disaster relief and resource allocation. A major ethical dilemma is that current rationing criteria may skew away from normally disadvantaged populations. In the past, allocation plans were developed by the healthcare community. In the current crisis, some planning groups have tried to address this by asking disparate communities throughout their region to offer input into the plans. 36

In all circumstances, rationing scarce medical resources is difficult and stressful. Such distribution, rather than being based on politics, money or power, must be based on an equitable (fair), openly available, pre-existing plan. It may be beneficial to have emergency physicians and intensivists take the lead (under set protocols) in making these decisions, since they have had more experience than others in doing this on a regular basis. Ideally, they will have support from their institutions’ bioethics consultants, social workers, and chaplains.

Rationing will not end when medications to treat COVID-19 are eventually identified or vaccines are produced for prevention. In the first weeks or months there will be limited amounts available, with massive public anguish over how they are being distributed. Those involved in developing and implementing healthcare resource distribution will need to think ahead and include this eventuality in their plans. Lastly, resource allocation is not the only option. Disasters are the exact situations where clinicians and administrators need to “think outside the box” by expanding clinical roles and responsibilities, relaxing restrictive regulations, improvising medical equipment, and devising other solutions to scarcity.^27^ Until the pandemic ends, we will need to encourage our healthcare workforce to stay at their posts and to use their fortitude and intellect as they face the multiple challenges involved with their jobs.

## CONCLUSION

- Physicians and other healthcare providers’ individual risks will vary by the nature of the causative agent, the provider’s activities and underlying health, and the protections offered and used.
- Provider and population perception of risk will probably not be congruent with reality.
- History and the nature of the healthcare professions demonstrate that we will have enough professional personnel to treat patients.
- History suggests that we will not have the necessary support personnel—unless we respect their jobs and their risks and communicate with them in an open and honest manner.
- The distribution of scarce, lifesaving resources will first require searching for alternatives and then making triage decisions based on careful planning with, if possible, widespread input.

## Figures and Tables

**Table 1:** Disaster responders’ concerns and planners’ potential mitigating actions.^5^,^18^–^25^

Responders’ Concerns	Mitigating Actions
Risk to/safety of responder	Actions to help protect responder: priority for vaccinations, priority for prophylactic/treatment medications, appropriate/sufficient PPE, and prespecified responder decontamination procedures Clear, continuous, consistent, honest, and transparent communication to all responders Continuously available (and updatated as necessary) disaster plan Knowledgeable individuals available to answer any workplace safety questions
Risk to/safety of responder’s family and loved ones	Actions to help protect family: priority for vaccinations, priority for prophylactic/treatment medications, decontaminating responder, and providing PPE at home Clear, proactive, consistent, honest, transparent, and ongoing communication from employer to responder’s family Continuously available (and updated as necessary) disaster plan Knowledgeable individuals available to answer any questions about responder and family safety
Child and elder care	Provide paid sitters or care at health care facility Arrange, in advance, for local governments to keep schools open, whenever possible
Risk to/safety of responder’s pets	Provide or pay for pet care
Trust/confidence in health care organization/leadership	Have and communicate to all employees an all-hazard disaster plan, including risk-reduction measures, that is easily accessible, practiced, and modified as necessary based on circumstances Maintain clear, continuous, consistent, honest, and transparent communication to all responders about current disaster knowledge and plan Overtly and continuously demonstrate duty to protect and support responders
Inadequate disaster-related Human Resource policies^30^	Provide life/disability insurance and liability/legal protection for duration of disaster response Responders may leave work as necessary Flexible work hours Clear return-to-work policies Provide responders with communication (if possible) to their families
Adequate reimbursement for time and activities	Guaranteed appropriate pay/comp time/bonus pay for level of their activities
Safe, guaranteed transportation	Private vans or rooms and board at health care facility Arrange, in advance, for local governments to keep mass transit systems running, whenever possible
Mandatory quarantine	Clear, consistent, and reasonable quarntine policy
Personal illness/PTSD	Guaranteed treatment for disaster-acquired medical/psychiatric problems
Job requirements	Effort to make all responders feel they are valued part of the disaster response Clear description of any modified job expectations/requirements during disaster

*PPE*, personal protective equippment; *PTSD*, postraumatic stress disorder.

Reprinted, with permission, from the *Journal of Environmental and Occupational Medicine*.^7^

**Table 2:** Strategies for Scarce Resource Situations.^27^–^30^

**Prepare**—e.g., anticipate challenges, develop plans, stockpile materials. Identify leaders who can source or develop alternative supplies and equipment. Identify and train risk communicators. Plan to mitigate personnel difficulties in responding. **Conserve**—implement conservation strategies for supplies in shortage or anticipated shortage to ensure the minimum impact/compromise possible (e.g., determining “at-risk” groups with priority for therapies in shortage and overall strategies to conserve use of oxygen delivery devices [i.e., ventilators] or PPE. **Substitute**—provide an equivalent or near-equivalent medication or delivery device. **Adapt**—use of equipment for alternative purposes (e.g., anesthesia machine as ventilator) **Re-use**—plan to re-use a wide variety of materials after appropriate disinfection or sterilization (e.g., may include oxygen delivery devices). **Re-allocate**—if no alternatives exist, remove a resource from one area/patient and allocate to another who has a higher likelihood of benefit (i.e., greater chance of surviving or more post-disease years to live).

*PPE*, personal protective equipment.
